# Plant growth improvement mediated by nitrate capture in co-composted biochar

**DOI:** 10.1038/srep11080

**Published:** 2015-06-09

**Authors:** Claudia I. Kammann, Hans-Peter Schmidt, Nicole Messerschmidt, Sebastian Linsel, Diedrich Steffens, Christoph Müller, Hans-Werner Koyro, Pellegrino Conte, Joseph Stephen

**Affiliations:** 1 Department of Plant Ecology, Justus-Liebig-University, Heinrich-Buff-Ring 26-32, 35392 Giessen, Germany; 2 Ithaka Institute for Carbon Intelligence, Ancienne Eglise 9, CH-1974 Arbaz, Switzerland; 3 Institute of Plant Nutrition, Justus-Liebig-University, Heinrich-Buff-Ring 26-32, 35392 Giessen, Germany; 4 Earth Science Institute, University College Dublin, Belfield, Dublin 4, Ireland; 5 Dipartimento di Scienze Agrarie e Forestali, Università degli Studi di Palermo, viale delle Scienze ed. 4 90128 - Palermo (Italy); 6 Discipline of Chemistry, University of Newcastle, Callaghan, NSW 2308, Australia; University of New South Wales, School of Material Science and Engineering, NSW 2052, Australia

## Abstract

Soil amendment with pyrogenic carbon (biochar) is discussed as strategy to improve soil fertility to enable economic plus environmental benefits. In temperate soils, however, the use of pure biochar mostly has moderately-negative to -positive yield effects. Here we demonstrate that co-composting considerably promoted biochars’ positive effects, largely by nitrate (nutrient) capture and delivery. In a full-factorial growth study with *Chenopodium quinoa*, biomass yield increased up to 305% in a sandy-poor soil amended with 2% (w/w) co-composted biochar (BC_comp_). Conversely, addition of 2% (w/w) untreated biochar (BC_pure_) decreased the biomass to 60% of the control. Growth-promoting (BC_comp_) as well as growth-reducing (BC_pure_) effects were more pronounced at lower nutrient-supply levels. Electro-ultra filtration and sequential biochar-particle washing revealed that co-composted biochar was nutrient-enriched, particularly with the anions nitrate and phosphate. The captured nitrate in BC_comp_ was (1) only partly detectable with standard methods, (2) largely protected against leaching, (3) partly plant-available, and (4) did not stimulate N_2_O emissions. We hypothesize that surface ageing plus non-conventional ion-water bonding in micro- and nano-pores promoted nitrate capture in biochar particles. Amending (N-rich) bio-waste with biochar may enhance its agronomic value and reduce nutrient losses from bio-wastes and agricultural soils.

With growing food, bio-energy and bio-material demands, new agricultural strategies are required to reduce the environmental costs of agricultural production^1^,^2^. The environmental ‘costs’ caused by extensive N fertilizer use include nitrate leaching, groundwater pollution, river, lake and coastal water eutrophication, and emissions of the potent greenhouse gas (GHG) nitrous oxide (N_2_O)^2^. Intensifying N fertilization to meet rising human demands may further increase the environmental costs. Thus, to mitigate global warming and adapt to future hazards (e.g. more massive rainfall events and severe droughts), agricultural practices are required that reduce N losses for a more effective N fertilizer use, and at the same time promote soil organic carbon (SOC) accumulation in soils^1^,^3^.

Biochar is a carbonaceous porous material obtained by pyrolysis of biomasses, and may offer the chance to adsorb and retain plant nutrients and improve soil fertility^4^. A number of benefits have been documented with biochar amendment to soils, including increased water holding capacity (WHC), reduced bulk density, liming, reduced N leaching, reduced N_2_O emissions, and others^5^,^6^,^7^,^8^,^9^,^10^,^11^. Agricultural yields were on average increased by 10%^12^,^13^ with a broad spectrum of responses, positive as well as negative. One problem is that desired benefits are unpredictable because the mechanisms of biochar action are poorly understood (e.g. N retention^14^). So far, when using large doses (>10 t ha^−1^ biochar) at once, the greatest positive plant responses were observed with mineral-rich biochars made from manure and straw, or when biochar amendment improved unfavorable soil conditions (e.g. water supply^6^,^9^,^15^,^16^; pH/liming^6^,^17^, heavy metal pollution^18^). When biochars age in soil their properties usually change^19^,^20^. Biochar surfaces interact with microorganisms, minerals, dissolved organic and inorganic compounds, roots, root exudates and gases^21^,^22^,^23^. The interactions can form nutrient-rich organo-mineral phases that are characterized by high concentrations of oxidized carbon species and probably nano-phase redox-active mineral oxides^21^,^24^ and surface-nutrient charging (see Supplementary Fig. S1).

The overall results from field trials with pure, untreated biochar are somewhat in contrast to traditional practices where biochar is used in mixtures of manures, human faeces, food waste and agricultural residues^25^ to increase agricultural yields in poor soils. Such practices have been recorded as long as 4500 years ago in the Amazon, in Australia or Germany, often resulting in Pretic dark soil horizons^26^,^27^,^28^. In Eastern Asia, the use of biochar-mineral blending to enhance aerobic and anaerobic composting has been practiced for many decades or probably centuries^25^, the later in a process often referred to as Bokashi-making. Thus, a way forward to improve the growth-promoting effects of biochars may be co-composting.

When it comes to biochar-composting two questions emerge: “How does biochar impact composting?”, and “How does composting impact biochar?” Experimental evidence is limited to a few studies. Most were carried out in Asian-pacific countries, investigating the effects of biochar addition when composting wet, nutrient-rich materials like manure and sewage sludge^29^,^30^,^31^,^32^,^33^,^34^,^35^,^36^,^37^,^38^,^39^. These studies indicate that biochar amendment during composting can (i) adjust the CN-ratio and serve as a bulking agent (replacing e.g. wood chips)^33^,^35^; (ii) improve the retention of N^32,34,39^; (iii) reduce the mobility of heavy metals (a positive effect if toxicity is reduced)^31^,^32^; (iv) increase the formation of stable humic compounds^35^,^38^, (v) suppress N_2_O emissions in sludge composting^36^, and (vi) change the microbial composition during the composting process^37^.

However, studies investigating the effect of composting on biochar properties are particularly scarce. Prost *et al.*^39^ showed that two different biochars, contained in meshbags and embedded in 1 m^3^ compost vessels, showed non-linear increases in the cation exchange capacity (CEC), water-extractable organic carbon and nutrient stocks in the biochar over time. The black carbon content of the biochar, a biochemical stability marker, remained unchanged; both biochars were not degraded^39^. However, a subsequent study to investigate the effects of these changes on plant growth was not carried out.

Based on biochar-composting results and the investigations of ancient fertile black earth soils we assumed that co-composting will considerably alter biochar, and improve its plant-growth promoting properties. Thus we hypothesize that co-composted biochar will enhance plant growth stronger than untreated biochar. Our aims were (1) to evaluate the effects of composting on the properties and effects of a woody biochar on plant growth; (2) to separate biochar effects from the effects of compost or mineral fertilizer addition; (3) to investigate if improvements in plant growth may come at the expense of unwanted environmental effects such as increased nitrate leaching, or higher N_2_O emissions after N fertilization; and finally, if positive effects are found, (4) to unravel mechanisms associated with the properties and nature of the co-composted biochar. The timely order of the work reported here was (i) the plant growth study as proof of concept, followed by (ii) investigations into alterations of biochar characteristics and functions caused by co-composting.

## Materials and Methods

### Biochar Production

The biochar was produced from woody chips (80% coniferous, 20% deciduous wood) by Carbon Terra (formerly German Charcoal GmbH) in vertical retorts. Pyrolysis treatment temperature was approx. 700 °C over a period of about 36 hours before passing a fire front at the end of the pyrolysis process. There, highest treatment temperatures of approx. 850 °C were reached for a short final period (biochar properties: Supplementary Table S1A). The initial biomass must have contained some adhering soil which was revealed by the examination of the biochar (BC_pure_) with scanning electron microscope (Supplementary Fig. S1), i.e. mineral phases on the carbon matrix must have been present before pyrolysis was carried out. Also, high concentrations of potentially redox active Fe and Mn phases on the sample were revealed (Supplementary Fig. S1). (A more detailed characterization of the biochar before and after composting is the subject of a further paper in preparation.) The biochar can be considered as a class 1 biochar as per the IBI standard (IBI = international biochar initiative), and premium quality as per the EBC standard (EBC = European biochar certificate); for biochar properties (BC_pure_) see Table S1A.

### Compost and biochar-compost production

Production of compost with and without biochar (BC) was carried out in three replicated windrows (2.50 × 10 m) in April-May 2011 at the Ithaka Institute (former Delinat Institute) in Valais, Switzerland^40^. The input materials consisted of animal manures, straw, rock powder, soil and mature compost (Supplementary Table S1B). The production followed the guidelines of aerobic quality composting^41^,^42^ with daily turnover of the compost windrows for two weeks, following turnover periods of three days for five more weeks^40^. To three out of six windrows, 20% (vol/vol) woody biochar (Supplementary Table S1A) was added at the start. After mixing, both variants quickly reached 60–70 °C; temperatures were significantly higher in the biochar-compost during the thermophilic phase. After 6 weeks the composts approached maturity. Pot trials with 8 different plant species during late-summer and autumn 2011 confirmed that both composts adhered to existing German/Swiss quality guidelines^43^ (plant biomass yields were on average 11% higher with biochar-compost than pure compost). The biochar-compost mostly had improved properties compared to the compost without biochar^40^ (either in tendency or significantly, e.g. preference by earthworms in the earthworm avoidance test, ISO-17512; significantly lower N_2_O emissions; Kammann *et al.*, book chapter in prep.).

To calculate the biochar content in the mature biochar-compost, the biochar content of the <5 mm sieved biochar-compost was determined by two methods using loss on ignition (n = 5 per mixture or sample): (i) against a calibration curve where pure compost had been mixed with increasing amounts of untreated pure biochar (particle size <5 mm), and (ii) by the calculations described by Koide *et al.*^44^. The results were not different (10.89% vs. 11.12% for methods (i) and (ii), respectively). Therefore we used an average BC-content in the BC-compost of 11% by weight in all calculations (see experimental set-up, Supplementary Fig. S2, and Supplementary methods for description of the biochar particle retrieval).

### Plant growth study with composted (BC_comp_) versus untreated (BC_pure_) biochar

To evaluate the effect of composting on biochar properties with a special focus on nitrogen and plant growth, a fully randomized three-factorial plant growth experiment was conducted. The pseudo-cereal *Chenopodium quinoa* Willd cv. Hualhuas was grown in a mixture of sandy loam, sand, and gravel (supplementary methods) in the greenhouse in pots (height 20 cm, diameter 10.4 cm^15^,^18^). The three factors were (1) ‘compost’, i.e. with/without compost addition (2% w/w), (2) ‘biochar treatment’ (no biochar = control (**ctrl**)); untreated biochar **(BC**_**pure**_, taken from the same biochar production charge that had been added in the composting); and co-composted biochar (**BC**_**comp**_; picked with forceps from the biochar-compost); and (3) ‘N-fertilization’ = low (N-28) and high (N-140) fertilization with a full-component liquid fertilizer (Table S2B). N-application amounts equaled 28 and 140 kg N ha^−1^ in 9 doses (dates and amounts see Supplementary Tables S2A, B). The mixtures were filled into the pots, and the initial amount of extractable mineral N was determined by 2 M KCl extraction (see below). Prior to sowing the water holding capacity (WHC) was determined by 24 h flooding plus 24 and 48 h drainage; the drainage water was collected and analyzed for mineral N. The total N_min_ loss was calculated and related to the initially KCl-extractable N_min_.

During cultivation the soil mixtures were daily adjusted to 65% of the maximum water holding capacity (WHC). For more detailed information see supplementary methods and Supplementary Figs. S2 and S3. The plants were kept in the vegetative-growth stage by repeatedly removing tiny emerging flowers to prevent seed formation, and thus termination of the vegetative growth (otherwise the weight difference between undernourished and well-nourished plants would have been even larger); biomass was harvested 81 days after sowing. The plants were divided into shoots and leaves without inflorescences; root biomass was retrieved by washing on day 82. Dry leaves were milled to powder and the C and N concentrations were measured by CN analyzer (VarioMax CNS macro-element analyzer, Elementar Analytical Systems GmbH, Hanau, Germany).

### Nutrient analyses of soil-biochar substrates, biochar particles and extracts

The produced compost and biochar-compost were analyzed by standardized methods in a commercial lab (Eurofins, Germany). Mineral nitrogen (i.e. NH_4_^+^, NO_3_^-^ also referred to as N_min_) and organic C and N concentrations (N_org_ = N_total_ - N_min_) were determined colorimetrically in water or KCl extracts with an autoanalyzer (Seal Analytical GmbH, Norderstedt, Germany)^45^. The following aqueous solutions were analyzed: (1) 2 M KCl extracts (1:2.5, g:ml) of the substrates after shaking one hour at 150 rpm on a horizontal rotary shaker and filtering; (2) filtered 2 M KCl extracts of repeatedly washed biochar particles (see below); (3) water leachates collected after the determination of the WHC; or (4) electro-ultra filtration (EUF) water extracts (see below). To investigate the degree of nutrient capture in BC_comp_, three sub-samples of each, BC_pure_ or BC_comp_ particles, were ball-milled and analyzed for their total nutrient contents with a CN analyzer (VarioMax Elementar, Hanau, Germany).

### Electro-Ultra filtration (EUF) and sequential washing of BC particles

BC_pure_ und BC_comp_ particles (plant study; method investigation below) were analyzed for their nutrient release characteristics as follows: During the first 30 min., 1 g of ball-milled biochar, mixed with 4 g of quartz sand, was extracted at 20 °C, 200 V and 15 mA; elutes were collected every 5 min. The sum of the extractable nutrients in fraction 1 represents the readily available plant nutrients^46^. The extraction was continued for another 30 min. at 80 °C, 400 V and 150 mA (fraction 2). Five minutes of extraction are considered to provide the potentially plant-deliverable nutrients^46^, but since the release of nutrients continued the extraction time was prolonged, with six elution fractions collected every 5 min. Extractable nutrients were analyzed by ICP-OES, or colorimetrically for N_min_, C_org_ and N_org_ via autoanalyzer as described above.

### Method comparison: Release of the biochar-captured nitrogen

BC particles of different particle size were again sieved and picked from the stored BC_comp_ compost, or sieved from BC_pure_ which had been stored in the same place/conditions than the biochar-compost (see Supplementary methods). Retrieved particle size classes were 2–5 mm (s1) (plant study: 3–5 mm); 5–6.2 mm (s2); and 6.2–8 mm (s3). Particle subsamples were extracted either by EUF as described above, or were sequentially washed. The aim was to understand (i) if the nitrate loading/release depends on BC-particle size, (ii) if the EUF method and the sequential washing will deliver the same total nitrate amount, and (iii) how much of the mineral N attached to the biochar may be easily released e.g. by percolating rainwater.

The sequential-washing procedure included shaking (150 rpm) of 1.5 g biochar with 10-ml extracting solution on a horizontal rotating shaker, subsequent filtering to separate filtrate and particles, then repeating and shaking/filtering the same particles again (1) 1 h with distilled water, (2) 24 h with new distilled water, (3) 1 h with 2 M KCl and (4) 24 h with new 2 M KCl. All filtrates were colorimetrically analyzed for mineral N (see above).

### Statistical Tests

Three-way ANOVA was used for the plant harvest data; two-way ANOVA was deployed for other data sets (WHC, N leaching etc.) where only two factors applied; the Tukey post-hoc test was employed to evaluate differences among biochar treatments. Results at p < 0.05 are considered significant. Shapiro-Wilk and Levene tests were used to ensure normal distribution and homogeneous variances. If normal distribution could not be achieved by transformations (e.g. N_2_O emissions) we tested each ‘biochar treatment’ subgroup separately by one-way ANOVA (Supplementary Fig. S6). All statistical testing was carried out with SigmaPlot vers. 12.0.3 (Systat Inc.).

## Results

### Water holding capacity and initial N retention

The addition of either compost or biochar significantly increased the WHC of the poor sandy soil mixture (Supplementary Fig. S3). BC_pure_ and BC_comp_ increased the WHC, and when applied together with compost, BC_comp_ was significantly better than BC_pure_, ranging between increases of 10 and 15%, respectively (Supplementary Fig. S3).

**Figure 1:**
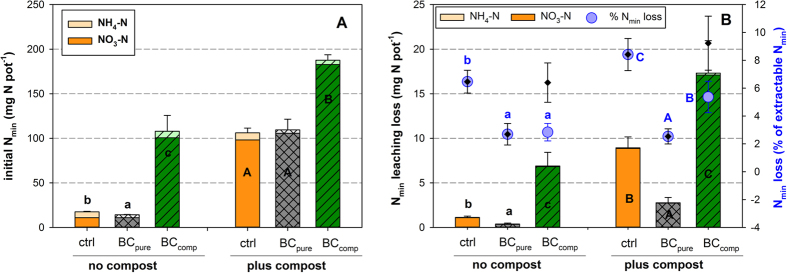
(**A**) Mean KCl-extractable N_min_ of the soil mixtures used in the plant study; (**B**) Mean N_min_ leaching loss with the drainage water after WHC determination (bars), or as relative N loss in % of the initially extractable N_min_ (small black diamonds), or in % of the initially extractable N_min_ plus BC_comp_-bound N_min_ (large blue dots); means ± s.d., n = 8. Black letters indicate significant differences (*p* < *0.05*) in the N_min_ content or -loss between biochar treatments, blue letters between %N losses (large blue dots) (two-way ANOVA and subsequent Tukey test).

The most abundant mineral N form in the initial soil mixtures was nitrate (Fig. 1A), and the KCl extraction already suggested that BC_comp_ carried and delivered nitrate (Fig. 1A; Table 1). The first leaching caused N_min_ losses between 2.5 and 9% of the initial KCl-extractable N_min_, with the largest absolute loss observed in the BC_comp_ treatment (Fig. 1B). However, when expressed in percent of the initial KCl-extractable N_min_ stocks, the relative N leaching loss in the BC_comp_ treatments with and without compost was equal to that of the respective controls (Fig. 1B). When the strong nitrate loading of the added BC_comp_ (see below) is included into the initial N_min_ stocks, the N_min_ leaching in both BC_comp_ treatments was significantly reduced compared to the controls (Fig. 1B). The addition of BC_pure_ always reduced the mineral N leaching significantly by 58% and 70% of the losses of the respective ‘no compost’ or ‘plus compost’ controls.

### Plant growth study

**Figure 2:**
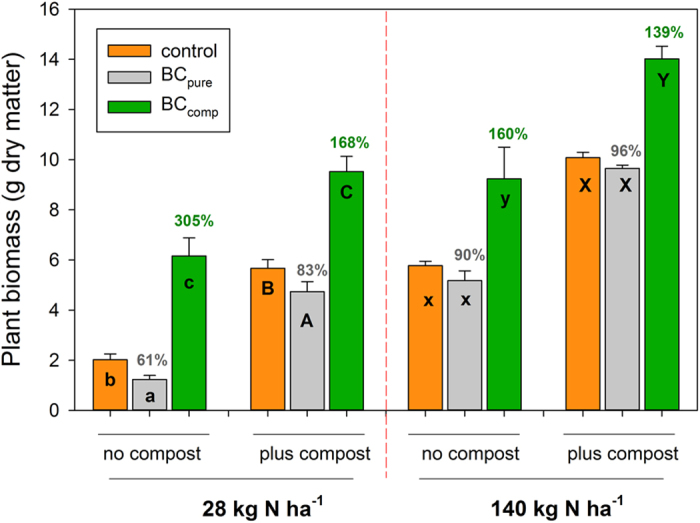
Total aboveground quinoa biomass at the final harvest (leaves and stems; mean + s.d., n = 4). Control (=no biochar); BC_pure_ untreated biochar; BC_comp_ composted biochar; ‘plus compost’, ‘no compost’: ±2% (w/w) compost addition; 28 and 140 kg N ha^-1^ indicate low and high full-compound fertilization, given by the cumulative N amount; different letters within a group of three bars indicate significant differences for ‘biochar treatment’ following three-way ANOVA (Tukey test, *p* < *0.05*), percentage values above bars show the relative change compared to the control (=100%) within the respective tree-bar group.

Compost addition and a higher fertilization significantly improved plant growth in all treatments (Fig. 2, Table 2). However, adding co-composted biochar always caused the largest plant growth increase (Fig. 2). The relative plant growth stimulation with BC_comp_, within the respective compost-fertilization treatment, was the stronger the lower the overall nutrient supply level was, ranging from 139% to 305% of the respective controls (Fig. 2). The absolute amount of biomass increase due to BC_comp_ addition was nearly identical at each nutrient-supply level (Fig. 2). Untreated biochar (BC_pure_) significantly reduced plant growth compared to the control in the N-28 treatment while in the N-140 treatments the reductions were not significant (Fig. 2). The amount of N taken up into the leaves closely mirrored the overall biomass results (Supplementary Fig. S5; all main factors and interactions: *p < 0.001*). The addition of 2% BC_comp_ produced the same amount of biomass and a higher N uptake into plant leaves than 2% compost (Fig. 2, Supplementary Fig. S5). The water use efficiency (WUE, the amount of water consumed per unit of plant biomass produced) was a function of the achieved plant size over time (not shown), confirming that the water supply had been optimal as intended. N_2_O emissions were low, below 12 μg N_2_O-N m^-1^ h^-1^, although they were measured after fertilizer addition (Supplementary Fig. S6). The ‘plus compost’ and ‘N-140’ treatments enhanced N_2_O emissions, compared to the lower nutrient supply levels. BC_comp_ addition did not stimulate N_2_O emissions above the respective controls in all nutrient supply levels (except for the lowest level), while BC_pure_ addition tended to (non-significantly) reduce N_2_O emissions (Supplementary Fig. S6).

### Composted biochar as a nutrient carrier

**Figure 3:**
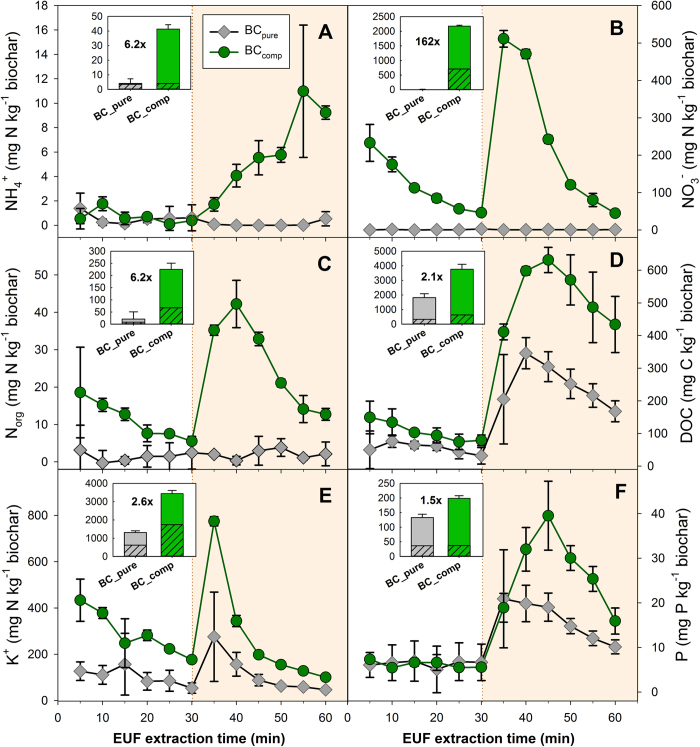
Nutrient release curves over time (means ± standard deviation, n = 3) of composted (BC_comp_) versus untreated biochar particles (BC_pure_; all particles 3 mm < particles < 5 mm) by electro-ultra filtration (EUF). The cumulative fraction 1 (first 30 min) equals plant-available nutrients; fraction 2 (shaded background, second 30 min.) equals potentially plant-deliverable nutrients (see methods). (**A**) ammonium; (**B**) nitrate; (**C**) organic N; (**D**) organic carbon; (**E**) potassium; (**F**) phosphorus. **Small inserts** show total extracted amounts, with the hatched bar part representing fraction 1, the non-hatched bar part fraction 2, with error bars (stdev.) given for the total cumulated sum (f1 + f2; n = 3). Numbers indicate the x-fold enhancement in the respective nutrient pool by co-composting.

**Figure 4:**
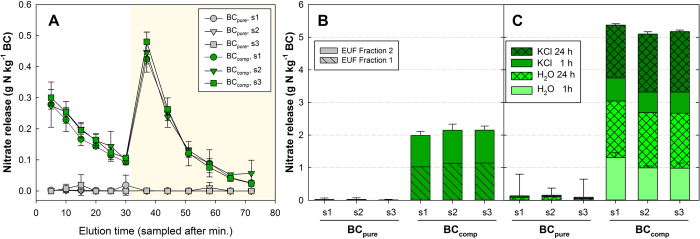
Nitrate release from untreated (BC_pure_) or co-composted (BC_comp_) biochar particles of three size classes (s1 = 2–5 mm; s2 = 5–6.2 mm; s3 = 6.2–8 mm), picked from three replicate biochar-compost samples (dots or bars: means ± standard deviation, n = 3). Methods: EUF extraction, (**A**) elution curves and (**B**) sums of fractions 1 (hatched) and 2 (plain); (**C**) sums of repeated washing with distilled water (H_2_O) and 2 M KCl (see methods). Error bars in (**C**) show the stdev. of the first release (1 h H_2_O washing, light green) and of the total sum.

**Figure 5:**
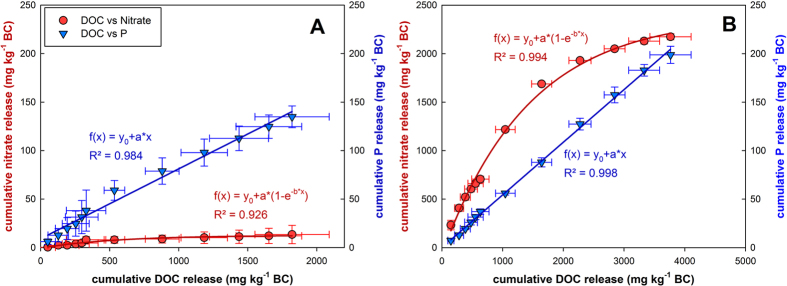
Correlation between the cumulative release of dissolved organic carbon (DOC) and nitrate (red dots); or between the cumulative release of DOC and phosphorus (blue triangles) over the course of the EUF extraction (Fig. 3). (**A**), release from BC_pure_; (**B**), release from BC_comp_; error bars are stdev. of the mean (n = 3); equations and regression analysis results are provided in the figure. Note that the phosphorus y-axes in A and B are similar, while the nitrate y-axis of B (BC_comp_) is 10-fold higher than the y-axis of A (BC_pure_).

The total N concentration of BC_comp_ particles was significantly higher than that of BC_pure_ particles (in percent: 1.04 ± 0.03 and 0.43 ± 0.06), i.e. composting had increased the total N concentration by 6.2 g N kg^-1^ biochar. Plant nutrient extractions using EUF revealed high nutrient contents on/in the BC_comp_ particles compared to BC_pure_ particles. Although NH_4_^+^was detected, the dominant mineral N form was nitrate, with more than 2000 mg NO_3_^−^ kg^−1^ (Fig. 3 A-C; Fig. 4A,B). The release dynamics of the dissolved organic carbon (DOC; Fig. 3D) were closely correlated to those of nitrate (exponentially, R^2^ = 0.994) or phosphate (linearly, R^2^ = 0.998; both *p < 0.001*, Fig. 5). For the anions nitrate and phosphate as well as DOC and N_org_, the larger absolute release occurred with fraction 2; only for K which is not well retained by biochar^47^ it occurred with fraction 1 (Fig. 3). Composting increased the level of cation loading for K^+^ 1.6-fold (Fig. 3E). Calcium (not shown) was increased by 63% from 3735 mg kg^−1^ in BC_pure_ to 6077 mg kg^−1^ in BC_comp_. In BC_pure_, magnesium was low (258 mg kg^−1^) and slightly reduced further to 156 mg kg by composting (not shown).

The sequential washing procedure revealed that only 40% of the total amount of nitrate and ammonium of BC_comp_ had been detected by EUF (Fig. 4B,C and Fig. 5B): BC_comp_ released in total 5214 mg nitrate-N kg^−1^ (mean all particle sizes), compared to the amount detected by EUF, 2174 mg nitrate-N kg^−1^ (Fig. 3, particle size 3–5 mm used in plant study) or 2095 mg nitrate-N kg^−1^ (mean of all particle sizes, Fig. 4B). Two-way analysis of variance indicated that ‘particle size’ had no impact, neither on the amount, nor timing, nor sums of the N_min_ release, regardless of the method that was used (Fig. 4). However ‘biochar type’ (i.e. BC_pure_ vs. BC_comp_) was always highly significant (*p < 0.001*).

## Discussion

The plant growth study confirmed our hypothesis that co-composting improved the biochars’ plant growth promoting effects. Plant growth was stimulated up to 5-fold with BC_comp_ compared to BC_pure_, and up to 3-fold compared to the control. Moreover, the positive effects were clearly related to the nutrient loading of the biochar. Nutrient loading was also observed by Prost *et al.*^39^ with two different co-composted biochars, i.e. mesh-bags filled with smaller-sized biochar particles were embedded in a model composting study. However, the nitrogen (nitrate) loading observed here was considerably larger^39^. The difference is likely due to the composting settings: we used N-rich manures in large-scale aerobic windrow composting, with daily and three-daily turnover (for oxygenation) and moisture control during the thermophilic and mesophilic phases. In addition the biochar particles were in direct contact with the decomposing biomass rather than in mesh bags. Since the rotting temperatures in the model composting only reached about 35 °C, the difference in the nitrate capture in our study and that of Prost *et al.* may be related to the higher temperatures (55–65 °C) during the thermophilic phase (see discussion below).

Untreated production-fresh biochar does not always have positive effects on plant growth, even when it is completely free of potential harmful toxic substances or heavy metals. Here, the untreated BC_pure_ had significantly *negative* effects on quinoa growth. However, neither a series of eco-toxicity tests nor chemical analyses revealed harmful substances in BC_pure_ beforehand; the biochar adhered in all measured parameters to the highest IBI or EBC standards.

Biochars can cause bi-phasic (or U-shaped/inversely U-shaped) plant responses^48^, at low concentrations often promoting and at high concentrations often suppressing plant growth or defenses to pests or pathogens^48^,^49^. Thus, adding 2% BC_pure_ may have shifted the bi-phasic plant response to the negative side^48^,^49^. However we consider it more likely that the negative BC_pure_ effect was primary caused by nitrate (and other nutrients’) capture, due to the following observations: (1) the growth reduction with BC_pure_ amendment was alleviated by increasing nutrient (nitrogen) supply of compost and/or fertilizer addition; (2) untreated BC_pure_ significantly reduced N_min_ leaching of both, ammonium and nitrate; (3) composting enhanced the nitrate concentration of the biochar far beyond its initial content, and far beyond the amount contained in the mature compost (>5000 mg nitrate-N kg^-1^, versus 980 mg nitrate-N kg^-1^), pointing towards very effective nitrate capturing mechanisms.

Nitrate capturing ‘symptoms’ have been observed in several studies (e.g. Prendergast-Miller *et al.*^50^,^51^, Haider *et al.*^16^ and others e.g.^7^,^52^,^53^). Nitrate retention (from aqueous solution, in much smaller amounts) was more pronounced with biochars produced at high (>600 °C) rather than low (<450 °C) temperatures^14^. Biochars’ anion exchange capacity is usually low, being 2-3 orders of magnitude lower compared to the cation exchange capacity^47^,^54^. Hence it was surprising that, in the present study, biochar captured considerable amounts of nitrate, was enriched in phosphate, and that both anions were not easily detached during EUF extraction (size of fraction 2 > fraction 1). We rather had expected repulsions between the negatively charged nitrate and the electron-rich biochar poly-aromatic material. Two mechanisms that may have contributed to nitrate capture on/in the porous biochar matrix are the development of acid and basic functional groups and organo-mineral complexes on the biochar-matrix surfaces^21^,^39^,^55^, and unconventional H-bonding (see below).

Composting clearly increased the DOC content of the biochar (Fig. 3D), which was also observed by Prost *et al.*^39^; furthermore its surface characteristics changed as expected due to composting (Supplementary Fig. S1). If more functional groups developed on BC_comp_ during composting (which was not investigated here) they may have been formed by oxidizing the biochar-matrix itself, by the attachment and uptake of macro-molecular organic compounds from the composted materials, or by both processes. It remains to be investigated if the observed exponential-rise-to-maximum type of correlation of nitrate-to-DOC release during the EUF extractions (Fig. 5) does have a mechanistic background, or if it is just coincidence.

Another mechanism for the observed nitrate capture may have been non-conventional water-ion hydrogen-bonding to the porous surface of the solid aging biochar matrix^56^,^57^. Again, this may or may not be interrelated with the dissolved organic carbon uptake/formation (Fig. 5). The interactions between water and the organic constituents of biochar can be obtained through the electron-donation from the π-clouds of the polyaromatic systems towards the electron-deficient hydrogens in water. This may lead to weak unconventional H-bonds between the asymmetrically shaped hydrated nitrate ions^58^ and the porous biochar surfaces. Moreover, Conte *et al.*^56^ recently showed that water movement in a pyrogenic poplar biochar was slow at temperatures up to 50 °C due to strong surface bonding forces; these forces considerably weakened at 80 °C. Subsequently the water movement within biochar changed with temperature, from a preferential 2D surface flow, to a 3D interaction of the bulk water in biochar nano-pores^56^. This is consistent with the large anion release (almost a “burst”) observed during the EUF when the temperature was raised from 20 °C to 80 °C (=EUF fraction 2, see Fig. 3B).

Conversely, the thermophilic phase (60–70 °C) during composting may have strengthened biochars’ nutrient capture capabilities through temperature-catalyzed inflow and H-bonding, in addition to the development of acid and base functional groups with compost ageing. Interestingly, nitrate release from BC_comp_ did not depend on biochar particle size, i.e. changing the surface-volume relationship did not have an impact. This warrants further investigations to identify the mechanisms.

The nitrate-/nutrient-capturing mechanisms observed here during composting may also occur when biochar ages in soils. Nutrient capture by biochar will depend on “opportunity”, i.e. encounters between e.g. nitrate/nutrient molecules in the soil solution and biochar particles; and on time, (milieu: soil or compost) temperature, pH, water content, and potentially alternating redox conditions^59^,^60^. Furthermore, it likely depends on the initial biochar properties such as the pore size distribution^56^,^57^, and acquired properties such as coating by organo-mineral complexes and redox active micro-sites (i.e. the availability of clay, Fe or Mn). Aerobic quality composting of biochar likely provides favorable conditions to enhance its properties for nitrate capture (thermophilic phase up to 70 °C, frequent alteration between oxic and anoxic conditions, clay and stone meal addition)^20^. In soils or lower-quality composting the same effects may also occur, but at a lower speed, in particular in dry or boreal soils^61^.

In any case, the observed nitrate/nutrient capture indicates that standard analytical methods using 1–2 hours of extraction/shaking will not detect all biochar-bound nutrients. Particularly nitrate may partly remain non-exchangeably captured, and hence may be frequently underestimated.

For future land-use schemes it is necessary to reduce the environmental ‘costs’ per unit of biomass/yield produced. During the initial WHC determination (first leaching), untreated BC_pure_ reduced nitrate leaching in absolute as well as relative terms. This is a well-known phenomenon of activated carbon (e.g.^62^) which has to some degree also been observed with biochars^7^,^53^,^63^,^64^. However the reduced nitrate loss in BC_pure_ treatments did not translate into improved plant growth, which may be better understandable now, in the light of the nitrate-capturing properties of the biochar used here. In contrast the N-enriched (composted) biochar promoted plant growth, but the initial nitrate leaching was higher in absolute terms. It did however not proportionally increase: based on the initial KCl-extraction results, BC_comp_-amended soils lost in relative terms the same percentage of nitrate by leaching than the controls. Moreover, when the entire extracted amount of captured nitrate is considered, the BC_comp_ treatments even retained more nitrate than the controls. During plant growth the nitrate load of BC_comp_ did not promote leaching: In the second leaching event, only marginal amounts of nitrate were leached with a simulated heavy rainfall (see Supplementary Methods and Results, and Supplementary Fig. S4). Interestingly, combining the nitrate-loaded BC_comp_ with compost was particularly powerful in retaining nitrate, compared to BC_comp_ addition alone (Supplementary Fig. S4), pointing to synergetic effect of biochar plus compost combinations in capturing, retaining and delivering N in plant-soil systems.

Theoretically, the nitrate- and DOC loading potential of BC_comp_ may also increase the potential for N_2_O production by creating denitrification “hot spots”^65^. Spokas *et al.*^66^ reported larger N_2_O emissions with field-aged biochar. This was not observed here with the nitrate-plus-DOC loaded BC_comp_ even after N fertilization. The untreated BC_pure_ amendment caused the well-known tendency towards N_2_O emission reduction (meta-study Cayuela *et al.*^5^) which was, however, not significant due to large variations in the control treatments. We hypothesize that nitrate and probably DOC capture in biochar may significantly contribute to the regularly observed N_2_O emission reduction with biochar amendments. We furthermore hypothesize that observed shifts in the denitrifier gene expression towards a more complete denitrification (NosZ genes^36^,^67^) may be connected to nitrate capture in biochar: Nitrate capture may either influence the ratio of electron donor to acceptor (labile organic carbon to nitrate) so that nosZ activity is promoted, lowering N_2_O/N_2_ ratios and increasing N_2_O reduction activity^68^,^69^; or DOC and nitrate capture in the biochar nano-pores (<10 nm) simply reduced the overall substrate availability to the denitrifying bacteria (~1 μm diameter). The result in both cases will likely be more complete denitrification to N_2_^36^,^67^,^69^. Moreover, Fe nano-particles observed on the biochar surfaces (Supplementary Fig. S1) may have supported the microbial Fe(II) oxidation via reduction of nitrate to N_2_^70^. Detailed investigations are necessary to evaluate these hypothetical mechanisms which may actually work in concert.

Taken together, we conclude that the nutrient-loaded BC_comp_ did promote plant growth at equal or reduced environmental ‘costs’, particularly in combination with compost.

Biochar was already proposed as valuable C-rich bulking agent when composting wet, nutrient-rich organic waste^32^,^33^,^34^,^35^. Its N retention capability was thought to be related (among other mechanisms) to reduced gaseous NH_3_ losses and NH_4_^+^adsorption^32^. However, the unexpected anion adsorption properties may be key for biochar-mediated long-term soil fertility increases by retaining mobile nutrients against leaching. Humus, regardless of how it may be defined^71^, follows a strict and universal C:N:P stoichiometry, and is preferentially built-up in the presence of this stoichiometry^72^. The DOC, nitrate and phosphate capture by biochar, and nitrate protection against leaching, may shift the C:N:P stoichiometry to more optimal conditions for humus formation on the pore-surface continuum of biochar. The formation of complex organic macro-molecules (humus) may even be promoted when a biochar is Fe- and Mn-nanoparticle-rich (as the one used here) due to “ferrous wheel” type reactions^73^, alongside microbial activities^24^,^60^. Liang *et al.*^74^ observed improved labile-C partitioning from decomposing plant litter to stable soil pools in Terra preta versus adjacent oxisol soils. We put forward the working hypothesis that production-fresh biochars may initially not be able to promote SOC formation (beside their own C persistence) until they are to some extent enriched in nutrients and additional non-biochar organic carbon. The biochar-organics interaction and nutrient capture observed with biochar composting in this study may therefore give first hints towards the mechanisms contributing to the development of fertile black-carbon rich soils^27^,^75^. More research is clearly needed to (i) elucidate the hypotheses given above (mechanistic understanding), and to (ii) enable the targeted production of “designer chars^54^” e.g. for nitrate capture purposes (application).

The practical guidance, however, for organic waste management may be to *“compost the organic (nutrient-rich) best, and pyrolyze the woody (nutrient-poor) rest”*. The economic success of biochar implementation will likely depend on value-generating cascading-usage strategies. Including smaller, repeated, and economically feasible additions of biochar in composting, biogas production, or in animal husbandry with its multitude of nutrient-rich organic wastes, may in the end turn “biochar revolution” into biochar (soil) evolution^76^, at a pace that is economically feasible, and more appropriate for soil formation processes.

## Additional Information

**How to cite this article**: Kammann, C. I. *et al.* Plant growth improvement mediated by nitrate capture in co-composted biochar. *Sci. Rep.* **5**, 11080; doi: 10.1038/srep11080 (2015).

## Supplementary Material

Supplementary Information

## Figures and Tables

**Table 1:** **Statistical results** a **of two-way ANOVAs of the initial soil mixture properties** b.

	*WHC (48* *h)*		*N*_*min_initial*_ *(KCl)*		*N*_*min_leached*_		*%N*_*leached.*_^c^	
*Factor*	*F*	*p*	*F*	*P*	*F*	*p*	*F*	*p*
Compost	336.8	***<0.001***	3327.2	***<0.001***	773.3	***<0.001***	32.7 (19.8)	***<0.001***
Biochar	626.0	***<0.001***	919.8	***<0.001***	523.3	***<0.001***	128.7 (93.4)	***<0.001***
Comp x BC	10.7	***<0.001***	696.5	***<0.001***	38.3	***<0.001***	10.5 (6.5)	***<0.003***

^a^Results correspond to Figure 1 and Supplementary Figure S3.

^b^Abbreviations: WHC, water holding capacity after 48 hours (Supplementary Fig. S3); N_min_initial_ (KCl), initially extractable amount of mineral N (Fig. 1A); N_min_leached_, amount of mineral N drained with the WHC determination (Fig. 1B); %N_leached_, amount of mineral N leached (WHC) in percent of the initial, KCl-detectable amount of N_min_ (Fig. 1B, large blue dots).

^c^F values in brackets refer to the diamonds in Fig. 1B (nitrate loading of BC_comp_ not considered).

**Table 2:** **Statistical results of three-way ANOVAs of the biomass data** a.

	*Abg. biomass*		*Leaves*		*Shoots*		*root:shoot*	
*Factor*	F	*p*	F	*p*	F	*p*	F	*p*
**Compost**	839.6	***<0.001***	438.9	***<0.001***	811.3	***<0.001***	22.2	***<0.001***
**Biochar**	376.2	***<0.001***	196.2	***<0.001***	388.4	***<0.001***	*0.079*	
**N-Fertil.**	895.1	***<0.001***	437.4	***<0.001***	889.1	***<0.001***	*0.780*	
**Comp. x BC**	41.8	***<0.001***	0.13	*0.876*	44.0	***<0.001***	3.24	*0.051*
**Comp. x N**	63.3	***<0.001***	9.55	***0.004***	75.7	***<0.001***	0.74	*0.397*
**BC x N**	62.3	***<0.001***	1.72	*0.193*	64.4	***<0.001***	0.34	*0.712*
**Comp x BC x N**	17.0	***<0.001***	0.13	*0.878*	19.4	***<0.001***	0.23	*0.799*

^a^Abbreviations: Abg biomass, aboveground (leaf plus shoot) biomass (Fig. 2); root:shoot, root:shoot ratio; Comp., factor ‘compost addition’; Biochar, BC, factor ‘biochar treatment’; N-Fertil., N, factor ‘low/high fertilization’.
