# Supplementary material for: Plant growth improvement mediated by nitrate capture in co-composted biochar
Source: Sci Rep. 2015 Jun 9;5:11080. doi: 10.1038/srep11080 (PMC4460888; doi:10.1038/srep11080)
Supplement: Supplementary Information [file srep11080-s1.doc]

Supplementary information to MS

Plant growth improvement mediated by nitrate capture in co-composted biochar

Claudia I. Kammann^1,2*^ Hans-Peter Schmidt^3^, Nicole Messerschmidt^1^, Sebastian Linsel^1^, Diedrich Steffens^4^, Christoph Müller^1,5^, Hans-Werner Koyro^1^, Pellegrino Conte^6^ & Stephen Joseph^7^

^1^Department of Plant Ecology, Justus-Liebig-University, Heinrich-Buff-Ring 26-32, 35392 Giessen, Germany.

^2^Current address: Hochschule Geisenheim University, Von-Lade Str. 1, D-65366 Geisenheim, Germany; *corresponding author: [Claudia.Kammann@hs-gm.de](mailto:Claudia.Kammann@hs-gm.de)

^3^Ithaka Institute for Carbon Intelligence, Ancienne Eglise 9, CH-1974 Arbaz, Switzerland

^4^Institute of Plant Nutrition, Justus-Liebig-University, Heinrich-Buff-Ring 26-32, 35392 Giessen, Germany.

^5^Earth Science Institute, University College Dublin, Belfield, Dublin 4, Ireland

^6^Dipartimento di Scienze Agrarie e Forestali, Università degli Studi di Palermo, viale delle Scienze ed. 4 90128 - Palermo (Italy)

^7^Discipline of Chemistry, University of Newcastle, Callaghan, NSW 2308, Australia; University of New South Wales, School of Material Science and Engineering, NSW 2052, Australia

*Please note that reference numbers given in this text partly refer to references listed in the main manuscript text.*

**Supplementary Information**

**The supplementary information encompasses the following more detailed descriptions and materials, given in the following order:**

Supplementary Materials and Methods:

- *Biochar and compost characterization and large-scale compost production*
- *Plant growth experiment: Preparation of the soil substrates*
- *Plant growth experiment: Experimental set-up and biochar particle retrieval*
- *Plant growth experiment: Determination of the water holding capacity and N leaching*
- *Plant growth experiment: Cultivation and harvest details*
- *Plant growth experiment: Nitrous oxide emissions after N fertilization*

Supplementary Results and Discussion Figures S1 and S5

Supplementary Tables S1A-C and S2A, B

- ***Table S1A****: Physical and chemical properties of the biochar*
- ***Table S1B****: Initial mixture of the composted materials*
- ***Table S1C****: Properties of the ripe composts (Eurofins Lab, Germany)*
- ***Table S2A****: Timing and applied sums of the liquid fertilizer*
- ***Table S2B****: Composition of the liquid Wuxal® fertilizer*

Supplementary Figures S1 – S8

- ***Figure S1****: Scanning electron microscopy pictures of BC_pure_ and BC_comp_*
- ***Figure S2****: Experimental set-up in graphical form, including BC particle sizes*
- ***Figure S3****: Water holding capacity of the different substrates*
- ***Figure S4****: Mineral-N loss after the 2^nd^ leaching (“rainstorm event”)*
- ***Figure S5****: Leaf total N uptake and relative N retrieval into leaves*
- ***Figure S6****: N_2_O emissions from Quinoa pots after the 2^nd^ partial N fertilization*
- ***Figure S7****: Photo of the control plants at the harvest date*
- ***Figure S8:*** *Photo of slightly magnified BC_pure_ particle versus BC_comp_ particle*

**Supplementary Materials and Methods**

*Biochar and compost characterization and large-scale compost production*

The biochar was produced from 80% varied hardwood and 20% varied coniferous wood chips. Pyrolysis took place in a "Schottdorf"-type reactor (Carbon Terra GmbH, Augsburg, Germany) at 750°C in a 36 hour cycle. The biochar had a pH of 9.5, a carbon content of 76% and H/C_org_ molar ratio of 0.2. Its specific surface (BET) area was 144 m^2^ g^-1^. Further physical and chemical properties of the biochar used in this trial are summarized in Tab. S1A.

The compost was produced following a standard protocol for professional aerobic quality composting (Kompostforum-Schweiz, 1998; Bernal et al., 2009^41^; Amlinger et al., 2008^42^). The composition of the initial mixture for composting is given in Table S1B. The piles were turned each day for the first five weeks and watered when necessary (daily water content determination). For the following three weeks, the piles were turned every three days. After eight weeks, when the pile core temperatures approached ambient values, the substrates were stored in 1.5 m^3^ open big bags. The substrates were investigated by ecotoxicology tests using (1) a closed-vessel cress test (Fuchs, 2000), (2) the barley germination and growth test (both taken from the German handbook of quality compost production^43^), (3) with 7 different plant growth tests (not shown; Kammann et al., book chapter in prep.) and (4) with the earthworm avoidance test ISO-17512 described in Busch et al. (2012)^77^, only that biochar-compost was directly compared to pure compost as the control. No harmful effects were found on animal survival, behavior or on plant germination and growth; the earthworms tended to prefer the biochar-compost over the pure-compost, and they preferred it significantly when the compost was amended with BC_pure_ before the test was carried out. The main characteristics of the ripe compost and biochar-compost are summarized in Table S1C.

*Microscopic biochar characterization before and after co-composting*

Samples were mounted on conductive tape and then coated with Chromium. Examination of the biochars was carried out using a Zeiss Sigma scanning electron microscope (SEM) fitted with a Bruker energy dispersive x-ray (EDS) analyzers as described in Joseph et al. (2013)^21^.

*Plant growth experiment: Preparation of the soil substrates*

A nutrient-poor sandy soil was artificially mixed from a commercial sandy loam, Lahn-sand (0-2 mm) and quartz gravel (2-8 mm) at a ratio of 30:50:20 percent (sources of supply: Quarzsandwerk Mittelhessen GmbH, Gießen and McMineral, Heuchelheim, Germany). The materials were previously not agriculturally used, and had not received fertilizer. For each pot (which also served as the bottom part of the GHG flux measurement chamber, see Kammann et al., 2011^15^ or Buss et al., 2012^18^) an amount of 1800 g dry soil mixture was used, and amended with the respective compost and biochar amounts (Supplementary Fig. S2). The pots had five small mesh-covered drainage holes (4 mm diameter) in their bottom cap.

*Plant growth experiment: Experimental set-up and biochar particle retrieval*

The experimental set-up of the three-factorial plant growth study is shown in Supplementary Fig. S2. For retrieving BC_comp_ particles they were picked with forceps from the biochar-compost as follows. The biochar-compost was first sieved to < 5mm to remove large objects (stones, woody reminder and other debris). Both composts were used in this form (i.e. < 5 mm). For particle picking, the biochar-compost was subsequently sieved through a 3-mm mesh sieve to remove all smaller particles. The BC_comp_ particles were then picked with forceps from the 3-mm sieve (i.e. the BC_comp_ particle size was 3-5 mm). The distinctive brittle ‘”feeling” when grabbing the particles with forceps (and occasionally breaking the particle if in doubt) ensured that no other dark materials were mistaken for biochar.

The untreated biochar (BC_pure_) was handled in the same way, i.e. it was also sieved through a 5-mm and then 3-mm sieve to obtain the same particle size fraction. Since the BC-compost (<5 mm) already contained 11% BC_comp_ (see below), this amount was included in the ‘BC_comp_ + compost’ treatment by using 2.22% BC-compost, and adding another 1.78% of hand-picked BC_comp_ particles (3-5 mm) until additions of 2% of both, compost and BC_comp_ particles, were achieved (Supplementary Fig. S2). Again, the same was done with the untreated BC_pure_ in the respective treatments: here, 2% of pure compost was blended with 0.22% BC_pure_ (particle size <5mm), and 1.78% of BC_pure_ with a particle size of 3-5 mm (Supplementary Fig. S2). By including the original BC-compost, we aimed at including the potentially altered microbiological community that may or may not have developed during the composting in the presence of biochar (which was not investigated). Per pot, 36 or 32.04 g BC_comp_ particles had to be had-picked to achieve the respective additions of 2% or 1.78%, respectively (Supplementary Fig. S2). Since the process was very time-consuming this limited the pot size (soil amount) that could be used.

*Plant growth experiment: Determination of the water holding capacity and N leaching*

The WHC of the potted soil mixtures was determined on those pots that were later used for growing the plants. Eight pots of each treatment were put in a large vessel and slowly flooded from the bottom up with water until the water level was above the soil surface, but 1 cm below the pot brim. The pots were covered with foil and wetted for 24 hours. Each pot was then put onto its own 2.5 L glass vessel (Weck®, Germany) to collect the drainage water for later analysis. The pots were covered with aluminum foil to prevent evaporation. Pots were weighed after 24 and 48 hours of drainage (Supplementary Fig. S3). The weight after 48 hours when dripping had completely stopped was used for calculating 100% WHC as the reference point for the cultivation: The WHC was kept at 65% of the maximum WHC of the respective treatment over the entire study. A second leaching event was conducted on day 49 after sowing. First the pot mixtures were adjusted to 65% WHC (daily adjustment). Subsequently, a rainstorm event was simulated where each pot received 212.3 ml, equivalent to 25 mm of immediate “rainfall”. The leachates were collected as described before. The volumes were determined; the solutions were filtered, and stored at 4°C until colorimetrically analyzed. The N leaching loss was negligible compared to the initial leaching (Fig. 1 versus Supplementary Fig. S4).

*Plant growth experiment: Cultivation and harvest details*

After determination of the WHC and completely randomized set-up of the pots in a controlled greenhouse chamber (day/night: 16/8 h and 20-24°C/14°C; relative humidity 60-70%), 5 pre-germinated, well-developed *Chenopodium quinoa* Willd. cv Hualhuas seedlings with visible radiculae were transferred to each pot. The randomization was done nearly every day after weighing the pots for the soil moisture adjustment.

To account for the growing above-ground plant fresh weight, additional “reserve” Quinoa plants were grown. These were harvested on day 44 after sowing, and a correlation between the aboveground fresh weight and leaf numbers were established, to enable accounting for the largest part of the fresh weight of the plants in the study by counting their leaves. (The plants grew quite differently due to the different treatments, see Fig. 2).

At the final harvest, one of the N-140 control plants differed considerably from all other Quinoa plants in the study (leaf size and plant habitus, see Photo S1). Therefore this plant was excluded from the final analyses.

During the course of the Quinoa growth experiment Wuxal® Super N-P-K liquid fertilizer (AGLUKON, Düsseldorf, Germany) was applied repeatedly with the daily watering (to achieve the respective WHC), summing up to total amounts of 28 and 140 kg N ha^-1^; timing and sums are listed in Table S2A, the composition of the fertilizer is given in Table S2B. On day 54, 10 ml of a 25 g/100 L micronutrient solution was applied to exclude the possibility of a developing iron deficiency. The micronutrient fertilizer (Ferty®, PLANTA Düngemittel GmbH, Regenstauf, Germany) contained 10% MgO, 0.5% B, 2% Cu-EDTA chelate, 3.5% Fe-HEDTA chelate, 0.5% Mn, 0.8% Mo and 0.3% Zn.

Due to the appearance of aphids and Thysanoptera, the following insecticides were applied: Universal-Schädlingsfrei (Bayer CropScience GmbH, Langenfeld, Germany) on days 23 and 28, and NeemAzal® - T/S (Trifolio-M GmbH, Lahnau, Germany) on days 45 and 63 after sowing, respectively.

All harvested living leaves per plant were dried, milled and analyzed with a CN analyzer (VarioMax, Elementar Analytical Systems GmbH, Hanau, Germany) for total N. Leaf-N uptake per pot was calculated based on leaf N concentration and dry weight (Supplementary Fig. S5, bars); since some senescent leaves that were shed overnight could not be attributed to their “pot of origin”, a complete N balance (plant N removal) was not attempted.

Leaf-N uptake into the leaf mass per pot was related to the KCl-extractable initial N_min_ amount in the soil substrate mixtures, *minus* the N_min_ amount that was leached during the initial WHC determination, *plus* the N amount applied with the fertilizer, and *plus* the theoretically deliverable N amount from the compost amendments (Supplementary Fig. S5, blue dots). For the latter, we assumed that, within the study duration, 10% of the organically-bound N in the compost was mineralized over time, and was delivered to the growing plants. We are aware that this assumption may not be entirely correct; and that biochar addition may have changed the N mineralization from the compost in either direction. Potential N delivery from the very poor sandy soil was not included. The calculation thus serves as a rough first assessment of the partitioning of the available N between the two compartments plant and soil.

*Plant growth experiment: Nitrous oxide emissions after N fertilization*

Nitrous oxide emissions were measured with the closed (dark) chamber technique as described earlier (Kammann et al. 2011)^15^ within one day after the second N-fertilizer application (see Table S2A). Three gas samples were taken at 0, 2 and 4 hours after chamber closure by syringe and analyzed within 24 hours on a gas chromatograph equipped with an FID (CH_4_) and ECD (N_2_O) using N_2_ as carrier gas. The GC was set up according to the scheme described by (Mosier and Mack, 1980)^78^ and equipped with an automated sampling unit (Loftfield et al. 1997)^79^. Gas fluxes were calculated by linear regression using the ideal gas law and based on the covered soil surface, chamber volume, air temperature and pressure during the cover. The methane fluxes (emission as well as uptake) were negligible in the artificially-mixed soil and are thus not reported.

**Supplementary Results and Discussion to Figures S1 and S5**

*Micron and Nanometer scale detectable changes in the biochar by co-composting*

Detailed analysis of the fresh and composted biochar was carried out using a range of techniques including high resolution Scanning Electron Microscopy, Field Emission Transmission Electron microscopy with Electron Energy Loss Spectroscopy and energy dispersive x-ray (EDS) analysis, Raman and FTIR spectroscopy, cyclic voltammetry, Boehm titration and fast field cycling (FFC) NMR relaxometry to help elucidate the changes that had occurred during the composting. From this analysis a more detailed discussion of mechanisms can be undertaken. This will be the subject of a second paper (manuscript in preparation).

*Leaf N uptake and N partitioning between plant and soil*

The leaf-N uptake reflected the biomass response to some extent, with two differences (compare patterns of Fig. 2 and Supplementary Fig. S5). First, BC_pure_ addition reduced the leaf-N uptake only in the treatment with the lowest N supply (N-28, no compost, Supplementary Fig. S5). Second, for biomass production, the ‘N-28 plus compost’ treatment resulted in the same biomass than the ‘N-140 no compost’ treatment (Fig. 2). However in the leaf-N uptake (Fig S5), the ‘N28 plus compost’ treatment resulted in significantly lower N uptake into leaves than the purely mineral ‘N-140 no compost’ treatment. Otherwise the BC_comp_ additions always increased the N uptake into the leaves, reflecting the increased leaf mass. In Supplementary Figure S5, from left to right, the leaf-N uptake with BC_comp_ was increased (in % of respective control) to 297%, 172%, 175% and 135%.

The largest absolute increase in the leaf-N uptake with BC_comp_ over that in the control (i.e. leaf-N BC_comp_ – control) was observed in the ‘no compost’ treatments (+25.2 mg N pot^-1^ and +30.1 mg N pot^-1^ in the N-28 and N-140 treatments, respectively). In both ‘plus compost’ treatments, this surplus amount was lower (+22.1 mg N pot^-1^ in both treatments, Supplementary Fig. S5.) Since it can be assumed that the added BC_comp_ will always have delivered the same amount of N (compare Fig. 4: s1 - s3), this may point to a compost-biochar interaction: relatively more N may have been retained in the biochar-compost-soil matrix, compared to the purely mineral-N fertilization. Consequently, the highest N use efficiency (leaf biomass produced per unit of leaf-N) was found in the control and BC_comp_ ‘N-28 plus compost’ treatments (not shown; Masterthesis Messerschmidt, 2013).

When the leaf-N uptake is expressed in percent of the soil-available N (blue dots), the uptake was roughly around 30% of the available N. Although the calculation may be somewhat error-prone with regard to compost-mineralization N delivery, the reducing effect of the BC_pure_ treatments is clearly visible. This may reflect the nitrate capturing behavior of the untreated (i.e. non-nitrate-preloaded) biochar. Thereby, more N may have been retained in the soil, but with the N being less available for plant uptake. We argue that negative effects on plant growth, occasionally reported with untreated but otherwise uncontaminated biochar^12^, may be related to the nitrate capturing ability observed in this study.

**Supplementary Tables**

**Supplementary Table S1A**. Analytical parameters of the biochar used in the composting experiment. Analytical methods followed the guidelines in the European Biochar Certificate (EBC, 2012); nutrient contents are total nutrients.

| Parameter | Unit | in fresh matter | in dry matter |
| --- | --- | --- | --- |
| Density | kg m^-3^ | 269 | - |
| Specific surface (BET) | m^-2^ g | 143.8 | - |
| Ash 550 °C | mass-% | 14.1 | 16.3 |
| Hydrogen | mass-% | 1.13 | 1.31 |
| Carbon | mass-% | 65.7 | 75.8 |
| Nitrogen | mass-% | 0.38 | 0.43 |
| Oxygen | mass-% | 5.3 | 6.2 |
| Carbonate CO_2_ | mass-% | 1.59 | 1.83 |
| Organic carbon | mass-% | 65.2 | 75.3 |
| H/C org. (molar) |  | 0.21 | 0.21 |
| O/C (molar) |  | 0.06 | 0.06 |
| pH |  | 9.5 | - |
| Electric conductivity | μS cm^-1^ | 578 | - |
| Salt content | g kg^-1^ | 2.85 | 3.28 |
| Phosphorous | mg kg^-1^ | - | 810 |
| Magnesium | mg kg^-1^ | - | 2580 |
| Calcium | mg kg^-1^ | - | 20500 |
| Potassium | mg kg^-1^ | - | 9030 |
| Natrium | mg kg^-1^ | - | 1700 |
| Iron | mg kg^-1^ | - | 8230 |
| Silicium | mg kg^-1^ | - | 35400 |
| Sulphur | mg kg^-1^ | - | 730 |

**Supplementary Table S1B.** Composition of the composts; for the biochar compost, 20 vol% woody biochar produced at 600-750 °C (German Charcoal GmbH) was added.

| Material for composting | Amount in mixture |
| --- | --- |
| Cow manure (with straw*) | 5.0  m³ |
| Horse manure (with saw dust*) | 0.6  m³ |
| Poultry manure | 0.1  m³ |
| Straw | 0.3  m³ |
| Soil | 0.6  m³ |
| Stone meal | 0.07 m³ |
| aerobic quality compost | 0.03 m³ |

*admixtures

**Supplementary Table S1C.** Analytical parameters of the compost and the biochar-compost. Analyses were carried out by Eurofins, Germany, following the guidelines of the Bundesgütegemeinschaft Kompost (Kehres 2006^43^); trace elements were analyzed according to DIN EN ISO 11885. DM, dry matter

|  | **Parameter** | **Unit** | **Compost without biochar**  (compost) | **Compost with biochar**  (BC-compost) |
| --- | --- | --- | --- | --- |
|  |  |  |  |  |
|  | Density | g l^-1^ DM | 770 | 620 |
|  | Dry matter | mass % FM | 79.8 | 74.6 |
|  | Organic matter | mass % DM | 27.8 | 33.2 |
| **Maturity** | Salt content | g KCl kg^-1^ DM | 13.4 | 13.7 |
|  | Total organic carbon (TOC) | mass % DM | 13.6 | 21.4 |
|  | C/N ratio | - | 13 | 17 |
|  | pH (1:10 CaCl_2_) | - | 7.2 | 7.2 |
|  | Nitrate-N (CaCl_2_) | g N t^-1^ DM | 1128 | 878 |
|  | Ammonium-N (CaCl_2_) | g N t^-1^ DM | 41 | 0.4 |
| **Macro-nutrients** | Total nitrogen (N_total_) | kg N t^-1^ DM | 12.8 | 12.1 |
|  | Mineral nitrogen (N_min_) | kg N t^-1^ DM | 1.17 | 0.891 |
|  | Phosphorus as P_2_O_5_ | mass % DM | 0.85 | 0.74 |
|  | Potassium as K_2_O | mass % DM | 1.1 | 1.18 |
|  | Magnesium (Mg) | mass % DM | 0.74 | 0.65 |
|  | Calcium (total; Ca) | mass % DM | 6.91 | 6.83 |
| **Trace elements** | Lead (Pb) | g t^-1^ DM | 15 | 15 |
|  | Cadmium (Cd) | g t^-1^ DM | <0.1 | <0.1 |
|  | Copper (Cu) | g t^-1^ DM | 36 | 32 |
|  | Nickel (Ni) | g t^-1^ DM | 25 | 24 |
|  | Zinc (Zn) | g t^-1^ DM | 130 | 130 |

**Supplementary Table S2A**. Days of liquid fertilizer application (days after sowing for pre-germination), amounts given as kg N ha^-1^; low-N sum: 28 kg N ha^-1^; high-N sum: 140 kg N ha^-1^. Plants were harvested at day 82.

| Day after sowing | low-N | high-N |  |
| --- | --- | --- | --- |
| 11 | 2 | 10 |  |
| 25 | 2 | 10 |  |
| 39 | 4 | 20 |  |
| 46 | 6 | 30 |  |
| 53 | 2 | 10 |  |
| 54 | 2 | 10 | (+micronutrient solution) |
| 55 | 2 | 10 |  |
| 66 | 4 | 20 |  |
| 73 | 4 | 20 |  |

**Supplementary Table S2B**. Wuxal® composition; *nutrient as EDTA chelate

| Plant nutrient | Wuxal composition (g L^-1^) |
| --- | --- |
| Total N | 100 |
| *thereof: NO_3_^-^-N* | *28.75* |
| *thereof: NH_4_^+^-N* | *46.35* |
| *thereof: Urea-N* | *25.00* |
| P_2_O_5_ | 100 |
| K_2_O | 75 |
| B | 0.12 |
| Cu* | 0.05 |
| Fe* | 0.25 |
| Mn* | 0.15 |
| Mo* | 0.01 |
| Zn* | 0.05 |

**Supplementary Figures S1 – S6**

**
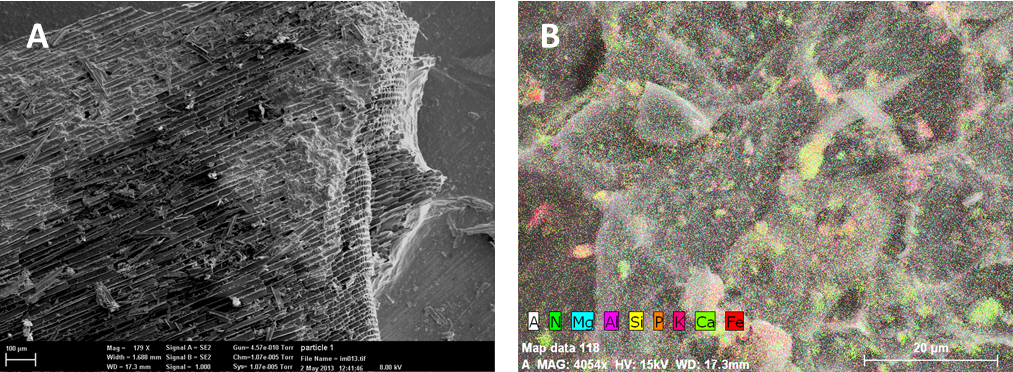
**

# Supplementary Figure S1. (A) Surface of the co-composted biochar, BC_comp_, with pores coated with a mixture of minerals, nutrients and organic matter after composting. (B) Heterogeneous distribution of minerals on the surface of BC_comp_ at high magnification. (C) Surface of the un-composted biochar, BC_pure_, with contamination of mineral matter. (D) BC_pure_ particle rich in Fe, P, Si and Al on the surface of the carbon matrix, indicting contamination of the original feedstock by some adhering soil before pyrolysis.

#

# Supplementary Figure S2. Set-up of the plant growth experiment with the three factors ‘*biochar treatment*’ (BC), ‘*compost addition*’ (no compost, plus compost) and ‘fertilization level’ (Low, N-28 and high, N-140) including the biochar particle size classes that were retrieved and used (compare Supplementary methods). ctrl = control, no biochar addition; BC_pure_ = untreated biochar; BC_comp_ = co-composted biochar

#

# Supplementary Figure S3. Water-holding capacity of the potted soil mixtures after 24 and 48 hours of drainage. Bars show means ± standard deviation (n=8); different letters indicate significant differences (one-way ANOVA) within each ‘*biochar treatment*’ group. Data were not normally distributed for the 24-h drainage results, thus one-way ANOVA was used for the ‘*no compost*’ or ‘*plus compost*’ groups; the two-way ANOVA of the 48-h drainage values (where normal distribution and equal variances were achieved) confirmed the same significant (*p<0.001*) effect of both factors ‘*biochar treatment,*’ and of ‘*compost*’, plus a highly significant ‘*biochar x compost*’ interaction (*p<0.001*).

**Supplementary Figure S4**. Mineral-N leaching loss during the second leaching (simulated strong rainfall event, see Supplementary methods). The entire bars show N_min_ means + s.d. (n=4), with nitrate (darker color) in the lower part and ammonium (lighter color) in the upper part.
**Note** **that** the N loss was more than 1000 fold lower than during the first leaching, compare Y-axis scales of Figure S4 and Figure 1; and that the combination of ‘BC_comp_ plus compost’ reduced any N_min_ leaching to virtually zero, compared to the ‘BC_comp_ without compost’ treatments. Three-way ANOVA analysis was not carried out due to lack of equal variances even with transformed data.

**Supplementary Figure S5**. Nitrogen uptake into (living) plant leaves per pot at the final harvest (bars), and leaf-N uptake expressed as percentage of the plant-available N (blue dots; calculation includes an assumption, see Supplementary methods). Error bars are standard deviations of the mean (n=4), different white or blue letters within the bars or above dots indicate significant differences between the respective biochar treatments (after three-way ANOVA); lower-case vs. uppercase letters indicate a significant compost effect, start- vs. end-alphabet letters indicate a significant fertilization effect. All main effects: *p<0.001*; Leaf-N uptake (bars): all interactions *p<0.001*; leaf-N in % of soil-N (dots): all interactions not significant.

#

# Supplementary Figure S6. Nitrous oxide emissions one day after the 2^nd^ application of mineral N fertilizer (=26 days after sowing; see Supplementary Table S2A), bars show means + standard deviation (n=4). Different letters within one ‘*biochar treatment*’ group (within a ‘*compost – N-fertilization*’ combination) indicate significant differences (*p<0.05*) by one-way ANOVA; normal distribution for three-way ANOVA was not achieved by transformations.

**Supplementary Figures 7 and 8: Photos**

#
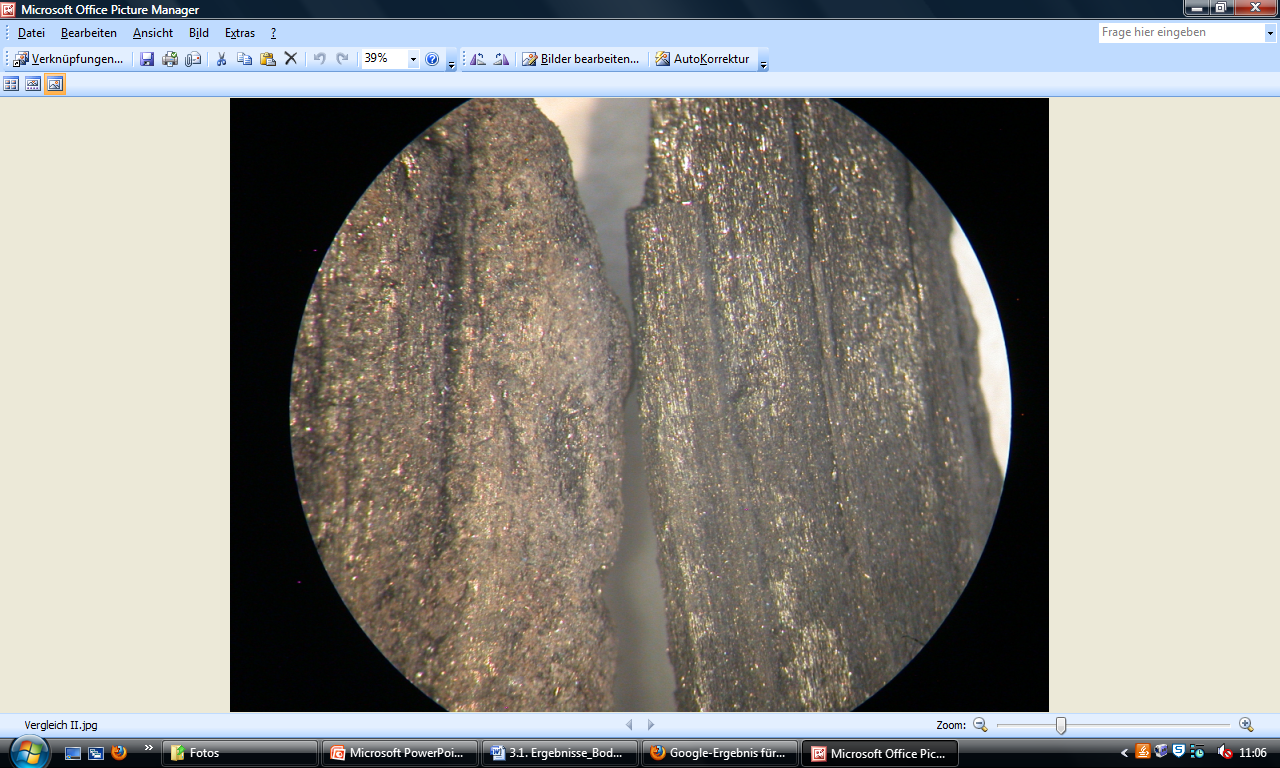


**Supplementary Figure S7**. Magnification of a BC_pure_ particle (left) and BC_comp_ particle (right); the co-composted biochar had a slightly more brownish and less glossy appearance. *Photo credit: N. Messerschmidt*


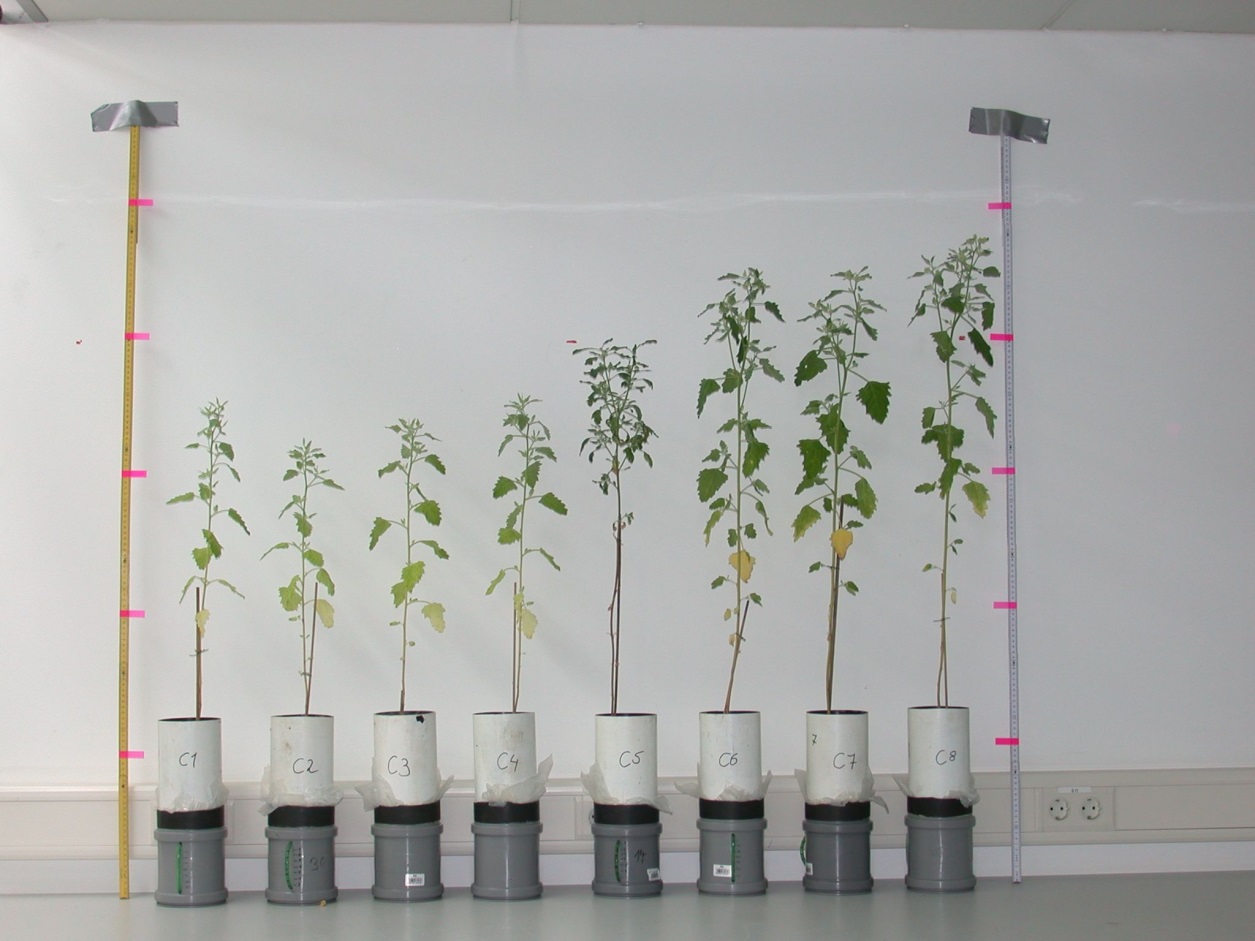


**Supplementary Figure S8**. Photo of the control quinoa plants at the day of harvest, left 4 plants N-28, and right 4 plants N-140. The habitus of the plant in pot C5 was clearly different and it was therefore excluded. *Photo credit: N. Messerschmidt*

**REFERENCES**

77 Busch, D., Kammann, C., Grünhage, L. & Müller, C. Simple biotoxicity tests for evaluation of carbonaceous soil additives: Establishment and reproducibility of four test procedures. *J. Environ. Qual.* **41**, 1023-1032 (2012).

78 Mosier, A. R. & Mack, L. Gas chromatographic system for precise, rapid analysis of nitrous oxide. *Soil Sci. Soc. Am. J.* **44**, 1121-1123 (1980).

79 Loftfield, N., Flessa, H., Augustin, J. & Beese, F. Automated gas chromatographic system for rapid analysis of the atmospheric trace gases methane, carbon dioxide, and nitrous oxide. *J. Environ. Qual.* **26**, 560-564 (1997).
